# Melatonin Regulates Apoptosis and Autophagy Via ROS-MST1 Pathway in Subarachnoid Hemorrhage

**DOI:** 10.3389/fnmol.2018.00093

**Published:** 2018-03-26

**Authors:** Ligen Shi, Feng Liang, Jingwei Zheng, Keren Zhou, Sheng Chen, Jun Yu, Jianmin Zhang

**Affiliations:** ^1^Department of Neurosurgery, Second Affiliated Hospital of Zhejiang University School of Medicine, Hangzhou, China; ^2^Brain Research Institute, Zhejiang University, Hangzhou, China; ^3^Collaborative Innovation Center for Brain Science, Zhejiang University, Hangzhou, China

**Keywords:** melatonin, MST1, apoptosis, autophagy, subarachnoid hemorrhage

## Abstract

Compelling evidence has indicated that imbalance between apoptosis and autophagy may be involved in subarachnoid hemorrhage (SAH). We aimed to investigate the effects and mechanisms of melatonin in the homeostasis of apoptosis and autophagy. One-hundred and forty-eight male Sprague-Dawley rats were intraperitoneally injected with melatonin or vehicle 2 h after SAH induction. Western blotting and an immunofluorescent assay were performed to detect the expression of apoptosis- and autophagy-related proteins. The neuroprotective effect of melatonin attenuating SAH-induced neurological deficit and brain edema may be associated with the suppression of SAH-induced neuronal apoptosis and autophagy. Furthermore, melatonin inhibited the cleavage of mammalian sterile 20-like kinase 1 (MST1) protein by reducing reactive oxygen species (ROS) content. These effects of melatonin on regulating the homeostasis between apoptosis and autophagy could be reversed by an MST1 agonist, chelerythrine, via enhancement of MST1 cleavage. In conclusion, exogenous melatonin alleviates SAH-induced early brain injury (EBI) by suppressing excessive neuronal apoptosis and autophagy. The underlying mechanism may, at least in part, involve the ROS-MST1 pathway.

## Introduction

Despite the tremendous progress made in intensive neuroprotective therapy and invasive surgical strategies, aneurysmal subarachnoid hemorrhage (SAH) remains a devastating disorder with a high risk of mortality and severe disability (Laiwalla et al., 2016; van Gijn et al., 2007). Early brain injury (EBI) occurs in the first 72 h following SAH, contributing to a poor prognosis (Cahill et al., 2006; Guo et al., 2017). Compelling evidence has indicated that an intrinsic apoptotic or autophagic cell death pathway is involved in EBI (Jing et al., 2012; Chen et al., 2014). Although an appealing hypothesis has been proposed that apoptosis and autophagy may be regulated by a common cellular factor (Dhingra and Kirshenbaum, 2013), no study has explored this missing link between apoptosis and autophagy in EBI after SAH.

Melatonin is an evolutionarily conserved neuro-hormone that is predominantly synthesized in and secreted from the pineal gland (Hu et al., 2017). Experimental evidence has demonstrated that both melatonin and its metabolites are potent free-radical scavengers and broad-spectrum antioxidants (Zhang et al., 2012), which maintain cellular homeostasis and survival by modulating inflammation, apoptosis, or autophagy following different types of brain injury (Fernández et al., 2015). Owing to its highly lipophilic nature, melatonin may be a promising agent that can easily pass through the blood–brain barrier (BBB) and play a neuroprotective role in various pathophysiological situations (Carloni et al., 2016). Previous investigators have reported that injection of melatonin after SAH induction reduced brain edema, decreased mortality and improved neurological function (Chen et al., 2014; Dong et al., 2016). At the molecular level, melatonin can inhibit neuronal apoptosis via up-regulation of the pro-survival protein Bcl-2 and down-regulation of its pro-apoptotic cognate Bax (Fernández et al., 2015). Furthermore, melatonin reduced the translocation of Bax to the mitochondria and inhibited mitochondria-dependent apoptosis by enhancing autophagy (Chen et al., 2014). However, no study has explored the detailed mechanisms underlying the regulation of apoptosis by melatonin-mediated autophagy.

Mammalian sterile 20-like kinase 1 (MST1) is a crucial serine-threonine kinase that constitutes a critical component of the Hippo signaling pathway (Chan et al., 2011). Compelling evidence has indicated that MST1 may be a switch that dually regulate apoptosis and autophagy (Dhingra and Kirshenbaum, 2013). In a crucial work, investigators showed that MST1 preferentially phosphorylates Beclin1 at Thr108 during ischemic stress in mouse hearts (Maejima et al., 2013). The phosphorylation of Beclin 1 impairs the formation of Beclin 1-Atg14L-Vps34, resulting in a marked reduction in autophagy (Maejima et al., 2013). In addition, the phosphorylated form of Beclin 1 can combine with Bcl-2 to destroy Bcl-2-Bax inhibitory complexes, resulting in an increase the amount of free cellular Bax available to induce mitochondria-dependent apoptosis (Maejima et al., 2013). In addition to the abovementioned mechanism, MST1 can be cleaved to produce a 36-kDa N-terminal constitutively active fragment (cl-MST1) during cell oxidative stress (Ura et al., 2001). Subsequently, the cl-MST1 is transferred into the nucleus and phosphorylates several histones, inducing neuronal cell apoptosis (Cheung et al., 2003). Interestingly, the enhancement of autophagy and reduction of apoptosis in response to melatonin can be reversed by blocking the MST1 pathway (Hu et al., 2017). Moreover, melatonin, as a potent free-radical scavenger, may regulate the activity of MST1 by scavenging reactive oxygen species (ROS; Lehtinen et al., 2006). However, the role of MST1 in melatonin-regulated apoptosis and autophagy in an SAH model has not been elucidated.

The present study was designed to verify the hypothesis that melatonin can regulate the homeostasis of apoptosis and autophagy by inhibiting the ROS-MST1 pathway in EBI after SAH induction.

## Materials and Methods

### Animals

All experimental animals were purchased from SLAC Laboratory Animal Company Limited (Shanghai, China). We used a total of 148 male Sprague-Dawley rats weighing 300–320 g. All rats were housed on 12-h/12-h light/dark cycles under temperature- and humidity-controlled conditions. Animal body temperature was maintained at 37°C. This study was carried out in accordance with the recommendations of the Guide for the Care and Use of Laboratory Animals of the National Institutes of Health and the Animal Research: Reporting of *in vivo* Experiments (ARRIVE) guidelines. The animal protocol was approved by the Institutional Ethics Committee of the Second Affiliated Hospital, Zhejiang University School of Medicine.

### SAH Model

The filament perforation model of SAH were performed according to previous studies (Marbacher, 2016). Briefly, after the rats were anesthetized with 40 mg/kg pentobarbital sodium via intraperitoneal injection, the left carotid artery and its branches were explored by microsurgical isolation. The distal left external carotid artery was transected and reflected in line with the ipsilateral internal carotid artery. A sharpened 4–0 nylon monofilament suture was placed lightly into the internal carotid artery from the ipsilateral external carotid artery and advanced through the internal carotid artery until the end of the suture reached the bifurcation of the anterior and middle cerebral arteries. The suture was advanced 1 mm to further to perforate the bifurcation of the anterior and middle cerebral arteries. After approximately 10 s, the suture was withdrawn. Sham rats received similar surgical procedures, but without perforation. All the experimental rats were maintained at 37°C using a heating pad throughout the surgical procedure.

The severity of the SAH was evaluated by a previously reported SAH grading system (Marbacher, 2016). The SAH grades represented the amount of bleeding in the subarachnoid space around the basilar artery rings and brainstem (Wu et al., 2016). Possible total scores range from 3 to 18, in which a higher score means more serious SAH. In the present study, we included only those rats with SAH grade scores ≥8 at 24 h after SAH induction.

### Drug Administration

Melatonin (N-acetyl-5-methoxytryptamine) was purchased from Sigma-Aldrich (St. Louis, MO, USA) and was dissolved in vehicle (1% ethanol in 1 mL saline) to create solutions for the doses of 5 mg/kg and 10 mg/kg. The melatonin and the vehicle (1% ethanol in 1 mL saline) were administered intraperitoneally at 2 h after SAH induction. Chelerythrine, an MST1 agonist, was dissolved in dimethyl sulfoxide (1 mmol/L) and administered immediately via intraventricular administration after SAH. Dimethyl sulfoxide was administered as vehicle control.

### Experimental Design

Two separate experiments were conducted in this study.

Experiment I was designed to assess the effect of melatonin on EBI after SAH induction and determine the optimal dose of melatonin for subsequent mechanistic experiments. The rats were randomly divided into four groups: the Sham group, the SAH+Vehicle (1% ethanol in 1 mL of saline) group, the SAH+Melatonin low-dose (5 mg/kg) group, and the SAH+Melatonin high-dose (10 mg/kg) group. Neurobehavioral functions, brain water content, brain ROS content, and neuronal apoptosis were examined at 24 h after SAH induction. Eighteen rats per group were applied to evaluate neurobehavioral functions, and then separately sacrificed to assess brain water content (*n* = 6 per group), brain ROS content (*n* = 6 per group), and neuronal apoptosis and autophagy (*n* = 6 per group).

Experiment II was designed to explore the molecular mechanism by which melatonin regulates the homeostasis of apoptosis and autophagy in EBI after SAH induction. The rats were randomly divided into four groups: the Sham+Vehicle (1% ethanol in 1 mL of saline, intraperitoneal administration; dimethyl sulfoxide, intraventricular injection) group, the SAH+Vehicle (1% ethanol in 1 mL of saline, intraperitoneal administration; dimethyl sulfoxide, intraventricular injection) group, the SAH+Melatonin (10 mg/kg, intraperitoneal administration) + Vehicle (dimethyl sulfoxide, intraventricular injection) group, and the SAH+Melatonin (10 mg/kg, intraperitoneal administration) + Chelerythrine (1 mmol/L, 10 μL, intraventricular injection) group. All rats were sacrificed at 24 h after SAH induction. Western blotting was performed to examine the expression of several target proteins including MST1, cl-MST1, Bcl-2, Bax, Beclin 1, LC3B-I, LC3B-II and cleaved-Caspase 3 (*n* = 6 per group), and immunofluorescence was used to detect apoptotic and autophagic neurons (*n* = 6 per group).

### Neurobehavioral Function Assessment

Neurobehavioral function was blindly assessed according to a modified Garcia scoring system as in previous studies (Marbacher, 2016). Briefly, this evaluation contains six test items: spontaneous activity (0–3), climbing (1–3), forelimb stretching (0–3), spontaneous movements of all limbs (0–3), body proprioception (1–3), and response to vibrissae touch (1–3). Possible scores range from 3 to 18, according to the severity of neurological deficits. A lower score means worse neurological deficits induced by SAH.

### Brain Edema Measurement

Intravital brain edema was examined by magnetic resonance imaging (MRI) as previously described (Guo et al., 2017; Dang et al., 2017). At 24 h after SAH induction, imaging was carried out in a 3.0-Tesla GE Discovery MR750 scanner (General Electric Company, Boston, MA, USA). T_2_ fast spin-echo sequences were taken using a field of view of 60 × 60 mm, a matrix of 256 × 256 mm and nine coronal slices (2 mm thick). All MRI data were analyzed by a blinded observer using NIH ImageJ software.

The degree of edema in the excised brain was measured as brain water content according to previous studies (Wu et al., 2016). After the rats were sacrificed at 24 h following SAH induction, their brains were removed and separated into the left hemisphere, right hemisphere, cerebellum, and brain stem. Each part was weighed (wet weight) and promptly dried for 72 h at 105°C (dry weight). The brain water content was defined as follows: brain water content = (wet weight − dry weight)/wet weight × 100%.

### Assay of ROS Expression

After SAH and sham rats were sacrificed at 24 h following endovascular perforation, brain tissue samples from around the basal cortical area on the injured side were obtained for assessment. Immediately, the total ROS level was measured with an ROS/RNS assay kit (Cell Biolabs Inc., San Diego, CA, USA), following the manufacturer’s instruction.

### Western Blotting

After SAH and sham rats were sacrificed at 24 h following endovascular perforation, brain tissue samples from around the basal cortical area on the injured side were obtained for assessment. Western blotting was performed as previously described (Pariente et al., 2016). Proteins were extracted from the brain tissue samples with RIPA buffer (Santa Cruz Biotechnology, Santa Cruz, CA, USA). The primary antibodies used were antibodies against MST1 (Cell Signaling Technology, Danvers, MA, USA, CST#3682), cl-MST1 (Cell Signaling Technology, CST#3682), active Caspase 3 (Abcam, ab49822), Bcl-2 (Cell Signaling Technology, CST#2876), Bax (Santa Cruz Biotechnology, SC-493), Beclin 1 (Proteintech, 11306-1-AP), and LC3B-I/II (Cell Signaling Technology, CST#4108).

### Immunofluorescence Staining

After 24 h following SAH induction, rats were perfused with PBS followed by 4% paraformaldehyde. The whole brain was immersed in 4% paraformaldehyde for 24 h, and then transferred to 30% sucrose solution for dehydration. Subsequently, the brain samples were frozen and cut into coronal slices (slice thickness: 8 μm) with a cryostat microtome (Leica CM3050S-3-1-1, Bannockburn, IL, USA). The sections were incubated overnight at 4°C with primary antibodies, including antibodies against NeuN (Abcam, ab177487 or ab104224) and LC3 (Cell Signaling Technology, CST#4108) followed by the appropriate fluorophore-conjugated secondary antibodies (Jackson ImmunoResearch, West Grove, PA, USA). TUNEL staining was performed according to the protocol from the manufacturer (Roche Inc., Switzerland) as described in previous studies (Ying et al., 2016). The sections were visualized with a fluorescence microscope (LSM-710; Zeiss, Oberkochen, Germany). The percentage of TUNEL-positive or LC3-positive neurons was calculated in a blinded manner.

### Statistical Analysis

All data were expressed as the mean ± standard deviation (SD). One-way ANOVA of the mean values followed by a Holm-Sidak test was performed for multiple groups. A Mann-Whitney U test was used for Garcia scores and SAH grading scores. The analyses were performed using SPSS version 22.0 (SPSS Inc.). Statistical significance was defined as *P* < 0.05.

## Results

### Overview of Body Weight, Blood Pressure, Temperature and Mortality in Both SAH and Sham Rats

During the experimental period, no significant difference was observed in body weight, mean arterial blood pressure, or temperature among the experimental groups (data not shown). The total mortality within 24 h after surgery was 14.19% (21 of 148 rats). Mortality showed no significant difference among SAH groups: 23.33% (7 of 30 rats) in the SAH+Vehicle group, 22.92% (11 of 48 rats) in the SAH+Melatonin group, and 25.00% (3 of 12 rats) in the SAH+Melatonin+Chelerythrine group. In addition, a total of seven SAH rats were excluded for insufficient bleeding (SAH grade <8).

### Melatonin Treatment Alleviated Brain Edema and Improved Neurological Function

**Figure 1:**
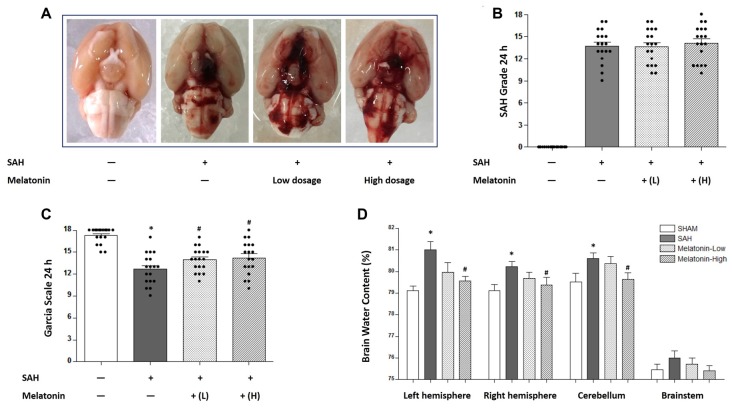
Both low and high doses of exogenous melatonin alleviated brain edema and improved neurological function 24 h after subarachnoid hemorrhage (SAH) induction. **(A)** The rats were randomly divided into four groups: the Sham group, the SAH+Vehicle (1% ethanol in 1 mL of saline) group, the SAH+Melatonin low-dose (5 mg/kg) group, and the SAH+Melatonin high-dose (10 mg/kg) group (*n* = 18 for each group). **(B)** SAH grade (*n* = 18 for each group): similar SAH grades were observed among SAH rats. **(C)** Garcia test (*n* = 18 for each group): garcia score decreased at 24 h after SAH induction. Both low and high doses of exogenous melatonin significantly increased Garcia scores compared with those of the SAH+Vehicle group. **(D)** Brain water content (*n* = 6 for each group): Low-dose melatonin treatment had no effect on brain water content. High-dose melatonin treatment significantly decreased brain water content in the left hemisphere, the right hemisphere, and the cerebellum. Error bars represented the mean ± standard deviation (SD). *P < 0.05 vs. the Sham group; ^#^*P* < 0.05 vs. the SAH+Vehicle group.

Two doses of melatonin (5 mg/kg and 10 mg/kg) were administered via intraperitoneal injection 2 h after SAH induction. No significant difference was observed in SAH scores among the SAH+Vehicle group (13.72 ± 2.244), the SAH+Melatonin low-dose group (13.61 ± 2.355), and the SAH+Melatonin high-dose group (14.11 ± 2.447) at 24 h following SAH induction (*P* > 0.05, Figures 1A,B). SAH rats receiving vehicle showed significantly worse neurological function than sham rats 24 h after SAH induction (Garcia scores: 12.67 ± 2.114 and 17.28 ± 1.074, respectively; *P* < 0.01; Figure 1C). Both low and high doses of melatonin therapy were associated with significant improvements in neurological function (Garcia score: 13.94 ± 1.626 for low-dose melatonin, 14.22 ± 2.290 for high-dose melatonin, vs. 12.67 ± 2.114 for SAH rats; *P* < 0.05; Figure 1C).

The degree of edema in each excised brain was measured as brain water content. SAH rats receiving vehicle showed elevated proportions of brain water content in the left hemisphere (81.01 ± 0.9270 vs. 79.11 ± 0.5128, *P* < 0.01; Figure 1D), the right hemisphere (80.22 ± 0.5824 vs. 79.12 ± 0.6885, *P* < 0.05; Figure 1D), and the cerebellum (80.61 ± 0.6580 vs.79.53 ± 0.9520, *P* < 0.05; Figure 1D) than sham rats. Low-dose melatonin showed no effect on reducing brain edema in any part of the rat brain (*P* > 0.05, Figure 1D). High-dose melatonin alleviated brain edema in the left hemisphere (79.56 ± 0.5259 vs. 81.01 ± 0.9270, *P* < 0.01; Figure 1D), the right hemisphere (79.21 ± 0.7091 vs. 80.22 ± 0.5824, *P* < 0.05; Figure 1D), and the cerebellum (79.65 ± 0.7151 vs. 80.61 ± 0.6580, *P* < 0.05; Figure 1D).

**Figure 2:**
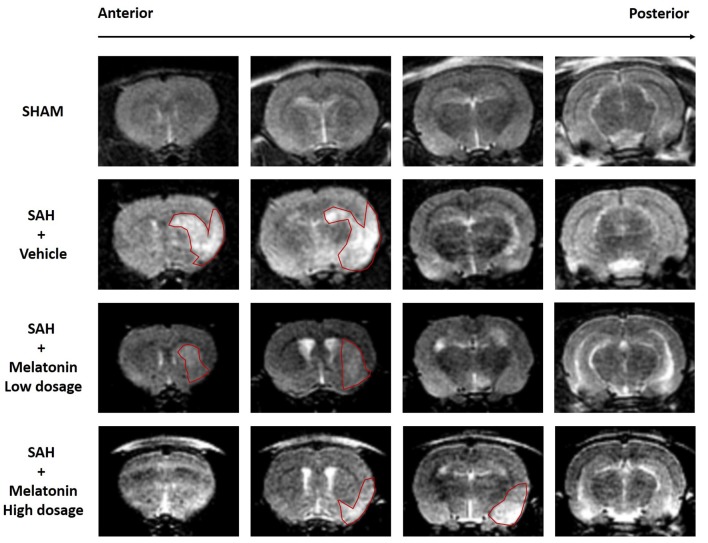
Both low and high doses of exogenous melatonin alleviated brain edema as measured by magnetic resonance imaging (MRI). Both low and high doses of melatonin, compared with vehicle, appeared to alleviate brain edema (red pane) in rats that had undergone SAH. In addition, SAH rats, regardless of whether they received vehicle or melatonin, had larger lateral ventricles than sham rats (*n* = 6 for each group).

Brain edema was assessed intravitally by MR imaging (Figure 2). SAH rats receiving vehicle showed a significant increase in brain edema compared with sham rats (Figure 2). Both low and high doses of melatonin appeared to alleviate brain edema compared with the amount in SAH rats receiving vehicle, but no statistical analysis was performed to test for this effect owing to the low resolution of 3-tesla MR scans (Figure 2). In addition, SAH rats, whether they received vehicle or melatonin, showed enlarged lateral ventricles compared to sham rats (Figure 2).

### Both Low and High Doses of Melatonin Treatment Inhibited Brain ROS Accumulation, Neuronal Apoptosis and Neuronal Autophagy

**Figure 3:**
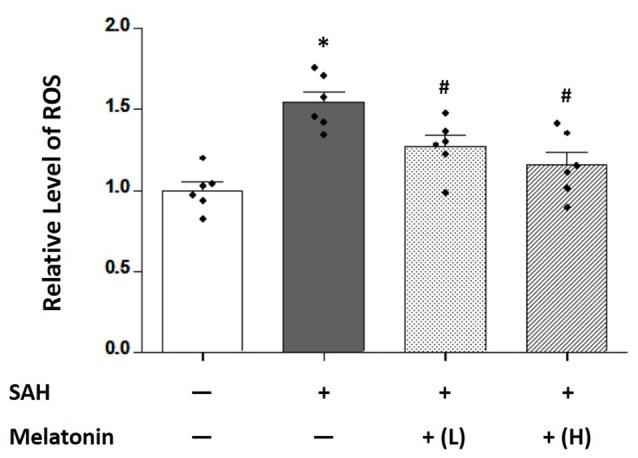
Both low and high doses of exogenous melatonin reduced SAH-induced reactive oxygen species (ROS) levels in brain tissues. Brain ROS content showed a significant increase in SAH rats receiving vehicle compared with sham rats. SAH rats receiving either low- or high-dose melatonin showed a significant inhibition of brain ROS accumulation (*n* = 6 for each group). Error bars represented the mean ± SD. *P < 0.05 vs. the Sham group; ^#^*P* < 0.05 vs. the SAH+Vehicle group.

Brain ROS content was significantly higher in SAH rats receiving vehicle than in sham rats (1.544 ± 0.1636 and 1.000 ± 0.1254, respectively; *P* < 0.01; Figure 3). SAH rats receiving either low- or high-dose melatonin showed a significant inhibition of brain ROS accumulation (1.272 ± 0.1659 for low-dose melatonin, 1.155 ± 0.1983 for high-dose melatonin, vs. 1.544 ± 0.1636 for SAH rats receiving vehicle; *P* < 0.05; Figure 3).

**Figure 4:**
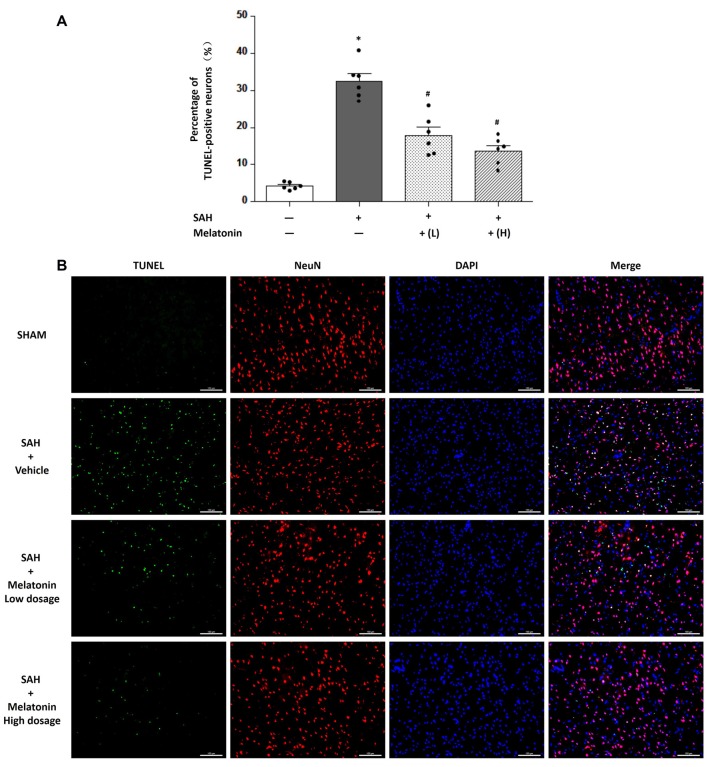
Both low and high doses of exogenous melatonin decreased neuronal cell apoptosis. **(A)** Neuronal cell apoptosis (*n* = 6 for each group): a significant increase in neuronal cell apoptosis was observed in the SAH+Vehicle group. Both low and high doses of melatonin treatment significantly decreased neuronal cell apoptosis compared with the level in the SAH+Vehicle group. Error bars represented the mean ± SD. *P < 0.05 vs. the Sham group; ^#^*P* < 0.05 vs. the SAH+Vehicle group. **(B)** Representative images of immunofluorescent staining show the TUNEL-positive neuronal cells (*n* = 6 for each group). Scale bar: 100 μm.

**Figure 5:**
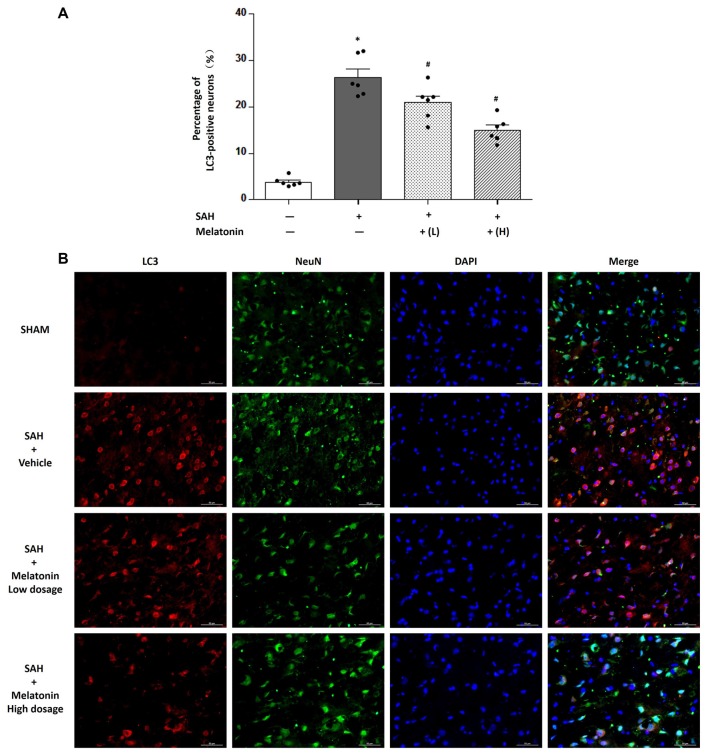
Both low and high doses of exogenous melatonin decreased neuronal cell autophagy. **(A)** Neuronal cell autophagy (*n* = 6 for each group): a significant increase in neuronal cell autophagy was observed in the SAH+Vehicle group. Both low and high doses of melatonin treatment significantly decreased neuronal cell autophagy compared with the level in the SAH+Vehicle group. Error bars represented the mean ± SD. *P < 0.05 vs. the Sham group; ^#^*P* < 0.05 vs. the SAH+Vehicle group. **(B)** Representative images of immunofluorescent staining show the LC3-positive neuronal cells (*n* = 6 for each group). Scale bar: 50 μm.

At 24 h after SAH induction, TUNEL staining was used to detect neuronal apoptosis, and LC3 staining was used to examine neuronal autophagy. SAH rats receiving vehicle showed higher percentage of TUNEL-positive (32.54 ± 4.877% vs. 4.203 ± 0.9677%, *P* < 0.01; Figure 4) and LC3-positive (26.36 ± 4.357% vs. 3.771 ± 1.016%, *P* < 0.01; Figure 5) neurons than sham rats did. SAH rats receiving either low- or high-dose melatonin showed significant inhibition of neuronal apoptosis (% of TUNEL-positive neurons: 17.95 ± 5.221% for low-dose melatonin, 13.74 ± 3.690% for high-dose melatonin, and 32.54 ± 4.877% for SAH rats receiving vehicle; *P* < 0.05; Figure 4) and neuronal autophagy (% of LC3-positive neurons: 20.92 ± 3.660% for low-dose melatonin, 14.97 ± 2.691% for high-dose melatonin, and 26.36 ± 4.357% for SAH rats, *P* < 0.05; Figure 5).

### Chelerythrine Abolished the Inhibitory Effects of Melatonin on SAH-Induced Apoptosis and Autophagy

**Figure 6:**
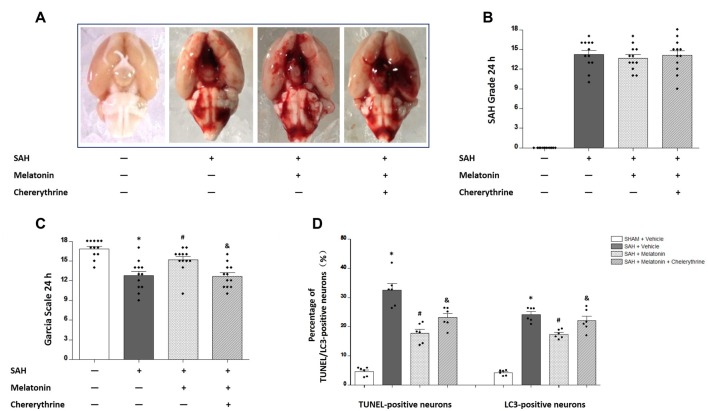
The effects of melatonin on SAH-induced brain injury were reversed by chelerythrine. **(A)** The rats were randomly divided into four groups: the Sham +Vehicle (1% ethanol in 1 mL of saline, intraperitoneal administration; dimethyl sulfoxide, intraventricular injection) group, the SAH+Vehicle (1% ethanol in 1 mL of saline, intraperitoneal administration; dimethyl sulfoxide, intraventricular injection) group, the SAH+Melatonin (10 mg/kg, intraperitoneal administration) + Vehicle (dimethyl sulfoxide, intraventricular injection) group, and the SAH+Melatonin (10 mg/kg, intraperitoneal administration) + Chelerythrine (1 mmol/L, 10 μL, intraventricular injection) group (*n* = 12 for each group). **(B)** SAH grade (*n* = 12 for each group): similar SAH grades were observed among SAH rats. **(C)** Garcia test (*n* = 12 for each group): Garcia scores were decreased 24 h after SAH induction. Melatonin could increase Garcia scores, and this effect was reversed by chelerythrine. **(D)** Percentage of TUNEL- or LC3-positive neurons (*n* = 12 for each group): SAH rats receiving vehicle showed a significant increase in TUNEL- and LC3-positive neurons compared with sham rats. Melatonin treatment could inhibit this SAH-induced neuronal apoptosis and autophagy; chelerythrine reversed this neuroprotective effect of melatonin on both apoptosis and autophagy. Error bars represented the mean ± SD. *P < 0.05 vs. the Sham group; ^#^*P* < 0.05 vs. the SAH+Vehicle group. ^&^*P* < 0.05 vs. the SAH+Melatonin group.

Chelerythrine is an MST1 agonist that promotes neuronal apoptosis by increasing caspase-dependent cleavage of MST1. No significant difference was observed in SAH grade scores among SAH rats receiving vehicle (14.25 ± 2.179), SAH rats receiving melatonin (13.67 ± 1.875), and SAH rats receiving melatonin plus chelerythrine (14.08 ± 2.539) at 24 h after SAH induction (*P* > 0.05, Figures 6A,B). SAH rats receiving vehicle had worse neurological function than sham rats (12.75 ± 2.261 vs. 16.83 ± 1.337, *P* < 0.01; Figure 6C); melatonin improved the SAH-induced neurologic deficit (14.58 ± 2.234 vs. 12.75 ± 2.261, *P* < 0.05; Figure 6C); and chelerythrine reversed this neuroprotective effect of melatonin (12.67 ± 1.826 vs. 14.58 ± 2.234, *P* < 0.05; Figure 6C). In addition, SAH rats receiving vehicle showed a significant increase in the percentages of TUNEL-positive (32.49 ± 5.601% vs. 4.623 ± 1.485%, *P* < 0.01; Figure 6D) and LC3-positive (24.13 ± 2.430% vs. 4.187 ± 0.9042%, *P* < 0.01; Figure 6D) neurons compared with sham rats; melatonin treatment inhibited this SAH-induced neuronal apoptosis (17.81 ± 3.304% vs. 32.49 ± 5.601%, *P* < 0.01; Figure 6D) and autophagy (17.41 ± 1.522% vs. 24.13 ± 2.430%, *P* < 0.01; Figure 6D); and chelerythrine reversed this neuroprotective effect of melatonin on both apoptosis (23.16 ± 3.425% vs. 17.81 ± 3.304%, *P* < 0.05; Figure 6D) and autophagy (22.06 ± 3.727% vs. 17.41 ± 1.522%, *P* < 0.05; Figure 6D).

**Figure 7:**
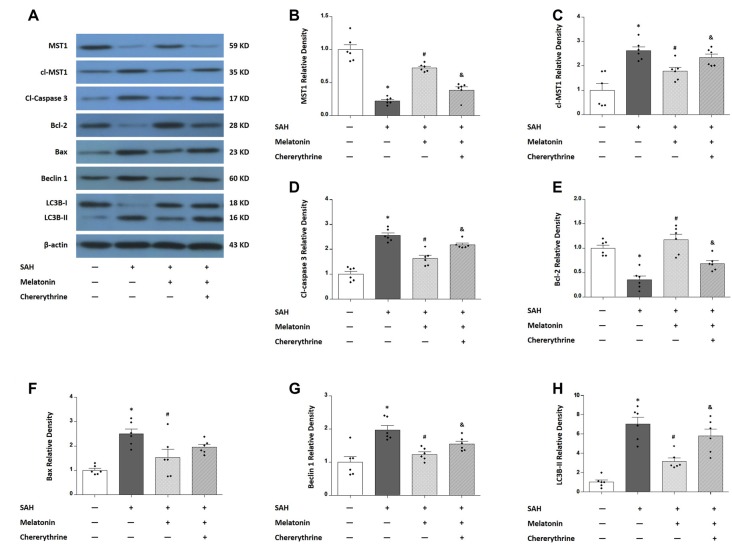
Western blots show the expression levels of mammalian sterile 20-like kinase 1 (MST1), cl-MST1, apoptosis-related proteins and autophagy-related proteins in response to melatonin treatment and chelerythrine administration. **(A)** Rats were randomly divided into four groups: the Sham group, the SAH+Vehicle group, the SAH+Melatonin group and the SAH+Melatonin+Chelerythrine group. SAH rats receiving vehicle showed the changes in the expression of MST1 **(B)**, cl-MST1 **(C)**, cleaved-Caspase 3 **(D)**, Bcl-2 **(E)**, Bax **(F)**, Beclin 1 **(G)** and LC3B-II **(H)**. Melatonin treatment attenuated the SAH-induced expressional changes in all these proteins **(B,H)**, and this effect of melatonin could be reversed by chelerythrine **(B,H)**. Error bars represented the mean ± SD. *P < 0.05 vs. the Sham group; ^#^*P* < 0.05 vs. the SAH+Vehicle group. ^&^*P* < 0.05 vs. the SAH+Melatonin group.

Western blotting was performed to measure the expression of MST1, cl-MST1, Bcl-2, Bax, cleaved-Caspase 3, Beclin 1, and LC3B-I/II (Figure 7). SAH rats receiving vehicle showed a 77.69% decrease in the expression of MST1 (*P* < 0.01, Figure 7B), a 262.1% increase in the expression of cl-MST1 (*P* < 0.01, Figure 7C), a 255.9% increase in the expression of cleaved-Caspase 3 (*P* < 0.01, Figure 7D), an 84.86% decrease in the expression of Bcl-2 (*P* < 0.01, Figure 7E), a 249.6% increase in the expression of Bax (*P* < 0.01, Figure 7F), a 197.5% increase in the expression of Beclin 1 (*P* < 0.01, Figure 7G), and a 326.5% increase in the expression of LC3B-II (*P* < 0.01, Figure 7H). Melatonin treatment decreased the expression of MST1 (0.7237 ± 0.05824 vs. 0.2231 ± 0.05328, *P* < 0.01; Figure 7B) while increasing the expression of cl-MST1 (1.7832 ± 0.3882 vs. 2.6212 ± 0.3957, *P* < 0.01; Figure 7C) compared with SAH rats receiving vehicle. In addition, melatonin therapy inhibited apoptosis-related proteins including cleaved-Caspase 3 (1.632 ± 0.2888 vs. 2.559 ± 0.2470, *P* < 0.01; Figure 7D) and Bax (1.535 ± 0.8086 vs. 2.496 ± 0.4967, *P* < 0.05; Figure 7F) while up-regulating the anti-apoptotic protein Bcl-2 (1.170 ± 0.2773 vs. 0.3514 ± 0.1897, *P* < 0.01; Figure 7E). Moreover, melatonin treatment inhibited autophagy-related parameters including Beclin 1 protein (1.2358 ± 0.1900 vs. 1.975 ± 0.3259, *P* < 0.01; Figure 7G) and the expression of LC3B-II (3.165 ± 0.3465 vs. 7.029 ± 06811, *P* < 0.05; Figure 7H). These effects of melatonin on regulating apoptosis and autophagy were reversed by chelerythrine, showing a significant increase in the expression of cl-MST1 (2.342 ± 0.3552 vs. 1.7832 ± 0.3882, *P* < 0.05; Figure 7C), cleaved-Caspase 3 (2.182 ± 0.1672 vs. 1.632 ± 0.2888, *P* < 0.01; Figure 7D), Belin 1 (1.544 ± 0.2099 vs. 1.2358 ± 0.1900, *P* < 0.05; Figure 7G), and the expression of LC3B-II (5.800 ± 0.7068 vs. 3.165 ± 0.3465, *P* < 0.05; Figure 7H), while there was a significant decrease in the expression of MST1 (0.3874 ± 0.1183 vs. 0.7237 ± 0.05824, *P* < 0.01; Figure 7B) and Bcl-2 (0.681 ± 0.1484 vs. 1.170 ± 0.2773, *P* < 0.01; Figure 7E).

## Discussion

The present study provided the first evidence that melatonin can regulate the homeostasis of apoptosis and autophagy. Melatonin showed neuroprotective effects by alleviating brain edema and improving neurological function. These properties were associated with the suppression of SAH-induced neuronal apoptosis and autophagy. The most inspiring finding in the present study was the potential mechanism in which melatonin might reduce brain ROS content to inhibit the cleavage of MST1 to produce cl-MST1, resulting in suppression of caspase-dependent apoptosis and Beclin 1-induced autophagy. In addition, the effect of melatonin on the expression of Bcl-2 and Bax might also be involved in its regulatory control over the balance between apoptosis and autophagy.

Melatonin provided strong protection for experimental SAH rats against EBI in the present study, alleviating brain edema, reducing ROS damage, and improving neurological function. These findings were consistent with previous studies reporting that either a low dose (5 or 10 mg/kg; Ersahin et al., 2009; Fang et al., 2009) or high dose melatonin (150 mg/kg; Wang et al., 2012a) via subarachnoid (Martinez-Cruz et al., 2002) or intraperitoneal injection (Ersahin et al., 2009; Wang et al., 2012a) could attenuate oxidative stress (Martinez-Cruz et al., 2002; Ersahin et al., 2009; Wang et al., 2012a), inhibit neuroinflammation (Fang et al., 2009), and reduce cell death (Wang et al., 2012a), resulting in reduction of mortality (Ersahin et al., 2009), alleviation of brain edema (Wang et al., 2012a), and amelioration of functional defects (Ersahin et al., 2009; Fang et al., 2009) in several different SAH models. Oxidative stress, largely generated by SAH-induced oxyhemoglobin stimulation, is the direct cause of cell death in the pathogenesis of EBI (Caner et al., 2012). Compelling evidence has indicated that melatonin, as a powerful free radical scavenger, may regulate several cell death pathways, including apoptosis and autophagy (Chen et al., 2014). Many studies have confirmed that melatonin can modulate the expression of the Bcl-2 family genes, producing up-regulation of the anti-apoptotic Bcl-2 and down-regulation of the pro-apoptotic Bax (Wu et al., 2017). These previous findings were confirmed by our present study, showing significant up-regulation of Bcl-2 and down-regulation of Bax in SAH rats receiving melatonin compared to SAH rats receiving vehicle. In addition, melatonin acts at complexes I and IV in the respiratory chain to promote mitochondrial oxidative phosphorylation, inhibiting intrinsic apoptosis in hepatic cells (Reiter et al., 2010).

However, the underlying mechanism whereby melatonin regulates autophagy remains largely unknown. Dual regulatory effects of melatonin on autophagy have been reported in previous studies, with both inhibitory and stimulatory roles for melatonin in autophagy (Eşrefoğlu et al., 2006; Motilva et al., 2011; Coto-Montes et al., 2012). Our present study indicated that melatonin (10 mg/kg via intraperitoneal injection) suppressed SAH-induced autophagy at 2 h after SAH induction. However, Chen et al. (2014) found that melatonin treatment (150 mg/kg, intraperitoneal injection) enhanced autophagy and decreased apoptotic cell death in the filament perforation model of SAH. These contradictory findings reflect the complexity of autophagy. Autophagy is a highly dynamic processes in which damaged cellular components are degraded (Levine and Klionsky, 2004). Enhanced autophagy can accelerate the clearance of damaged organelles or cytosol, thus promoting cell survival (Graef and Nunnari, 2011). However, it should be noted that autophagy is a stress-responsive process that is highly activated under extreme conditions (Wang et al., 2012b). We believed that melatonin improved the intercellular environment through several actions, including attenuating oxidative stress (Martinez-Cruz et al., 2002; Ersahin et al., 2009; Wang et al., 2012a), inhibiting neuroinflammation (Fang et al., 2009), and reducing neuronal apoptosis (Wang et al., 2012a). Thus, melatonin down-regulated autophagy in response to the improvement of the intercellular environment.

**Figure 8:**
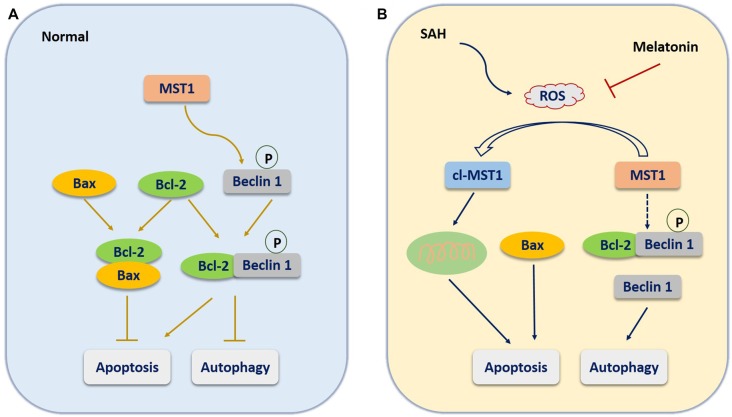
The molecular mechanism potentially involved in the role of melatonin in regulating the balance between apoptosis and autophagy. **(A)** Under normal conditions, Bcl-2 binds with Bax to inhibit the Bax-induced formation of a channel that is permeable to cytochrome *c* to activate caspase-dependent apoptosis. In addition, MST1 present in the cell at baseline helps maintain cell survival by phosphorylating Beclin 1 to prevent the formation of Beclin 1-Atg14L-Vps34, which would inhibit autophagy and activate apoptosis. **(B)** When SAH occurs, the increased brain ROS content activates the cleavage of MST1 to produce cl-MST1, which is transferred into the nucleus and phosphorylates several histones, inducing neuronal cell apoptosis. In addition, SAH stress reduces the expression of Bcl-2 protein, resulting in the displacement of Bax from Bcl-2 to increase apoptosis. Meanwhile, both the decrease in Beclin 1 phosphorylation by MST1 and the reduced expression of Bcl-2 cause the dissociation of the Bcl-2/Beclin 1 complex, enhancing cell autophagy.

The most inspiring aspect of the present study was that it explored the regulatory mechanism controlling apoptosis vs. autophagy. The cellular kinase MST1 may be a critical molecule that regulates apoptosis and autophagy by altering the expression or association of Bcl-2, Bax, and Beclin 1 (Dhingra and Kirshenbaum, 2013). Under normal conditions (Figure 8A), Bcl-2 binds with Bax to inhibit the Bax-induced formation of a channel that is permeable to cytochrome *c* to activate caspase-dependent apoptosis (Cahill et al., 2007). In addition, MST1 present in the cell at baseline helps maintain cell survival by phosphorylating Beclin 1 to prevent the formation of Beclin 1-Atg14L-Vps34, which would inhibit autophagy and activate apoptosis (Maejima et al., 2013). When SAH occurs (Figure 8B), the increased brain ROS content activates the cleavage of MST1 to produce cl-MST1, which is transferred into the nucleus and phosphorylates several histones, leading to neuronal cell apoptosis (Cheung et al., 2003). In addition, SAH stress reduced the expression of Bcl-2 protein, with the result that Bax was displaced from Bcl-2 to increase apoptosis. Meanwhile, both the decreased phosphorylation of Beclin 1 by MST1 and the reduced expression of Bcl-2 caused dissociation of the Bcl-2/Beclin 1 complex, enhancing cell autophagy (Dhingra and Kirshenbaum, 2013). Consistent with this theory, we found that the expression of cl-MST1, Bax, cleaved-Caspase 3, Beclin 1 and LC3B-II were dramatically increased, while the expression of MST1 and Bcl-2 was significantly decreased in rats after SAH. Melatonin showed its powerful free-radical scavenging capacity by reducing SAH-induced brain ROS accumulation, leading to suppression of MST1 to maintain the balance between apoptosis and autophagy.

Several limitations should be noted in the present study. First, we did not examine the interactions among Bcl-2, Bax, and Beclin 1, although the combinations of Bcl-2/Bax and Bcl-2/Beclin 1 were examined in previous studies (Youle and Strasser, 2008; Maejima et al., 2013). Second, the present study focused only on the role of melatonin in the ROS-MST1 pathway. Although the effects of melatonin on regulating apoptosis and autophagy can be attenuated by chelerythrine, other molecular pathways may also participate in this regulatory action of melatonin. Finally, autophagy is a dynamic and complex process including three sequential steps: formation of autophagosomes, fusion of autophagosomes with lysosomes, and degradation (Galluzzi et al., 2016). The increased protein levels of LC3II can be observed in both formation and degradation stages (Galluzzi et al., 2016). Although the present study didn’t provide direct evidence to distinguish these two stages, previous studies indicated that formation of autophagosomes is predominated rather than degradation in the first 24 h after SAH (Lee et al., 2009).

In conclusion, the present study provides the first evidence that melatonin alleviates SAH-induced brain injury including brain edema and functional defects by suppressing excessive neuronal apoptosis and autophagy. The underlying mechanism may, at least in part, involve the ROS-MST1 pathway. Further studies must explore the long-term effect of melatonin on SAH-induced brain injury by other potential pathways.

## Author Contributions

JianminZ was the principal investigator. LS and SC designed the study and developed the analysis plan. LS, FL, JingweiZ and JY completed this experiment. FL and JingweiZ analyzed the data. LS and FL contributed to writing the article. KZ revised the manuscript and SC polished the language.

## Conflict of Interest Statement

The authors declare that the research was conducted in the absence of any commercial or financial relationships that could be construed as a potential conflict of interest.
